# The relationship between MHC−peptide interaction and resistance to virus in chickens

**DOI:** 10.1002/iid3.596

**Published:** 2022-02-10

**Authors:** Yuan‐chang Jin, Yu‐feng Li, Li‐xia Jiang, Wei Wang, Chuan‐dan Zheng, Ming‐li Chen, Yu‐jie Wu, Juan Dai, Jing‐fen Chen, Min‐min Yu, Gang Zeng, Mei‐lin Hao, Bo‐ping Zeng

**Affiliations:** ^1^ Characteristic Laboratory of Animal Resources Conservation and Utilization of Chishui River Basin, Department of Biology and Agriculture Zunyi Normal College Zunyi People's Republic of China; ^2^ College of Agriculture and Food Engineering Baise University Baise People's Republic of China; ^3^ School of Life Science Hunan University of Science and Technology Xiangtan People's Republic of China

**Keywords:** antigen presentation, chicken, MHC I‐related molecules, virus resistance

## Abstract

**Introduction:**

The MHC‐peptide interaction has a subtle influence on host resistance to virus. This paper aims to study the relationship between MHC‐peptide interaction and MHC‐related virus‐resistance.

**Methods:**

By 3D homology modeling, the structure of chicken BF2 molecule BF2*0201 (PDB code: 4d0d) was studied and compared with the known structures of BF2 molecule BF2*0401 (PDB code: 4e0r) to elucidate the characteristics of BF2*0201‐binding antigenic peptides.

**Results:**

The results show that due to the amino acid difference between the two binding groove of 4e0r and 4d0d, the size of the binding groove of the two are 1130 Å³ and1380 Å³ respectively, indicating the amino acid species that 4e0r binding peptide has lower selectivity than 4d0d; and because of large side chain conformation of Arg (especially Arg111) of 4e0r replaced by small side chain Tyr111 of 4d0d, the volume of central part of the binding groove of 4d0d is obviously larger than that of 4e0r, indicating that the restrictive of binding antigenic peptides for 4d0d is narrower than that of 4e0r; and on account of the chargeability of the binding groove of the two are different, namely the binding groove chargeability of 4e0r (strong positive polarity) and 4d0d (weak negative polarity).

**Conclusion:**

There are generally more peptides presented by the BF2 of B2 haplotype than by that of B4 haplotype, leading to more resistance of B2 than that of B4 to virus.

## Graphical abstract

The MHC−peptide interaction has a subtle influence on host resistance to the virus. This paper aims to study the relationship between MHC−peptide interaction and MHC‐related virus resistance

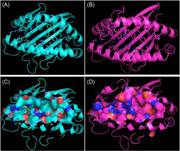

## 1 INTRODUCTION

With particular traits across all jawed vertebrates, the major histocompatibility complex (MHC) has two glycoproteins of primary sorts binding peptides that came from antigens of intracellular or extracellular to present to circulating T cells and have an integral effect on immune systems of innate and adaptive.^1^

MHC I, which provides the circumstance in which the pathogen is identified, primarily defines the specificity of adaptive immune responses. The *BF* genes, *MHC I* of chickens, contain two classes of *Ia* genes: *BF1* (previously designated *BFI*, *BFII*, or *BF* minor), *BF2* (also called *BFIV* or *BF* major) genes. Before the discovery of the *BF2* gene as a single gene presenting chicken classical peptide antigen, considerable headway has been made in showing the configuration of the BF2 and displaying its function.^2^, ^3^

The *BF2* gene in the chicken has been assumed to be responsible for the MHC‐related Rous Sarcoma (RS) or Marek's Disease (MD) resistance in chickens.^4^, ^5^, ^6^

B21 haplotypes are generally reported to confer the strongest resistance to MD, B2 medium resistance, and B4 weak resistance.^4^, ^7^, ^8^ For RS, B21 and B2 haplotypes have stronger resistance than B4.^5^, ^9^, ^10^

The objective of this study was to study the structure of BF2 molecule BF2*0201 of the B2 haplotype, which was compared with that of the B4 to elucidate the characteristics of BF2*0201‐binding antigenic peptides, and with the characteristics of BF2*2101‐binding antigenic peptides in previous literature,^11^ to further investigate the relationship between MHC−peptide interaction and resistance to virus in chickens.

## 2 MATERIALS AND METHODS

### 2.1 Comparison of amino acid sequences of 4d0d/4e0r

The amino acid (AA) sequences of 4d0d (BF2*0201‐VL8) and 4e0r (BF2*0401‐IE8) were procured from Protein Data Bank (PDB) respectively, which include A chain, B chain, and C chain. For fasta files of 4e0r AA sequences, DNAMAN version 5.0 software was used to compare α1−α2 superdomain of A chain with that of 4d0d amino acid sequences.

### 2.2 Comparison of DSSP of 4d0d/4e0r

The definition of the secondary structure of proteins (DSSP) of 4d0d/4e0r was procured from the PDB website. The differences of DSSP between 4d0d/4e0r on A chain, B chain, and C chain were analyzed.

### 2.3 Comparison of carbon backbone of 4d0d/4e0r

The carbon backbone of 4d0d/4e0r was compared, and the root mean square error (RMSD) value of which was compared.

### 2.4 Comparison of peptide binding grooves of 4d0d/4e0r

Structural figures and electrostatic potential surfaces of 4d0d/4e0r were obtained by the 3D molecular model (PyMOL) version 1.7 software molecular graphics system, and they were compared visually with each other under the same magnification. By using PyMol v1.7 software, the amino acids of peptide binding grooves were eventually identified. The comparison of 4d0d/4e0r in properties of the pocket of the binding grooves was well described in previous literature.^11^

### 2.5 Comparison of the mode of interaction between peptide and binding grooves of 4d0d/4e0r

The comparison of 4d0d with 4e0r of differences of the 3D modes of interaction between peptide residue and pocket was carried out using the PyMol v1.7 software.

## 3 RESULTS

### 3.1 General structure of 4d0d/4e0r

**Figure 1:**
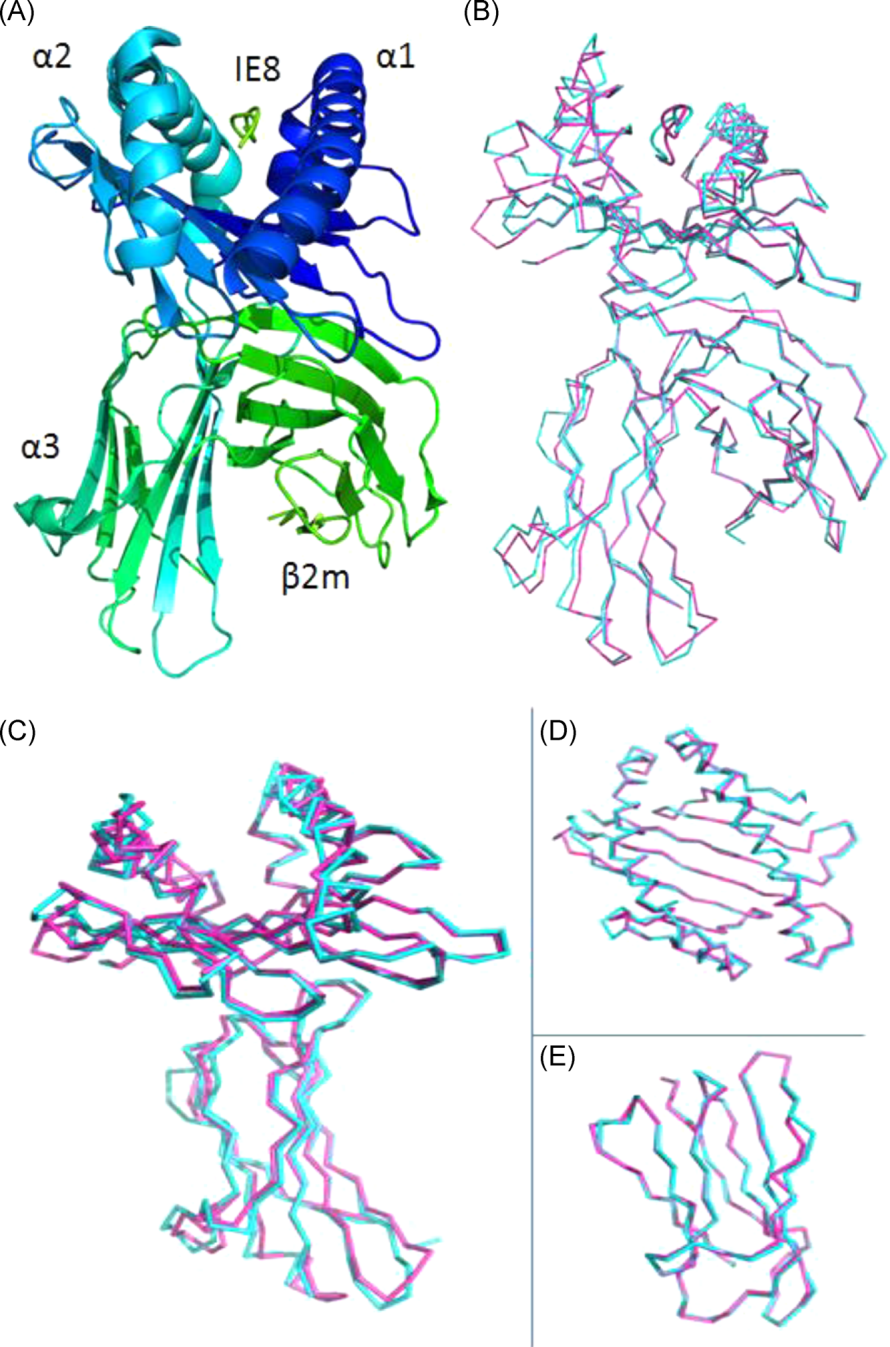
The structure of the chicken MHC I molecule BF2*0401(PDB code: 4e0r) and its comparison with BF2*0201(PDB code: 4d0d). (A) The overall structure of chicken BF2*0401, a typical MHCI structure. (B) Superimposed Cα‐traces of chicken BF2*0201‐VL8 (blue), BF2*0401‐IE8 (purple). It shows that the overall structures of the two chickens MHC I are very similar. (C−E) Carbon backbone deviation analysis of BF2*0201‐VL8 and BF2*0401‐ IE8. (C) The ratio of the carbon backbone of the A chain. (D) The ratio of the carbon backbone of the α1‐α2 chain. (E) The ratio of the carbon backbone of the B chain

**Figure 2:**
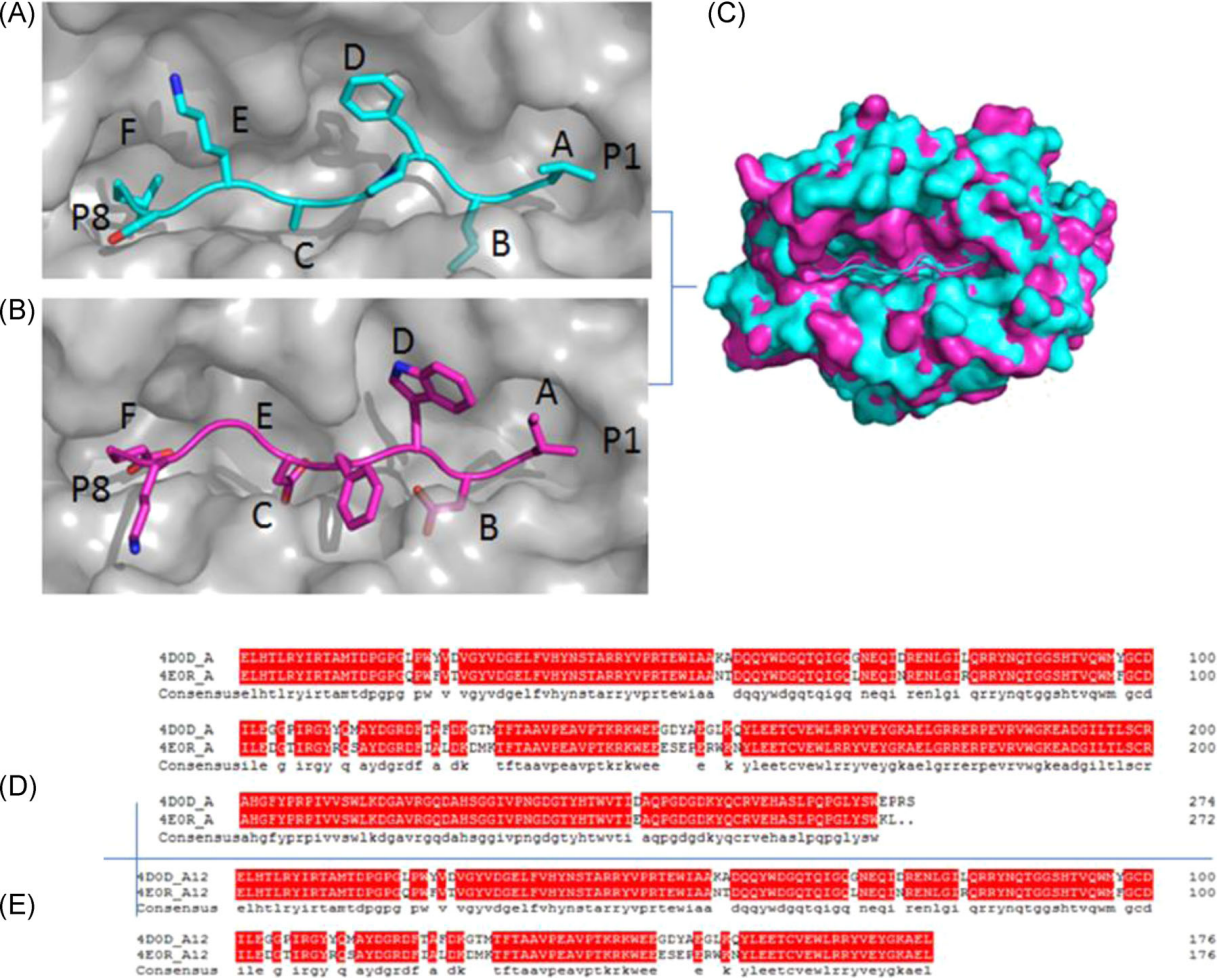
The peptide‐binding groove of BF2*0201and its comparison with BF2*0401. Comparison of the peptide‐binding grooves between BF2*0201 and BF2*0401, illuminating a relatively large binding groove of BF2*0201 and an extremely narrow groove of BF2*0401. (A, B) Molecular surfaces (gray) of the peptide‐binding grooves of BF2*0201 (A), BF2*0401 (B) with the peptides (VL8, IE8 colored in blue and purple, respectively). The N‐(P1) and C‐(P8) termini of the peptides are marked. Pockets in each groove are sequentially labeled A−F. (C) The surface of the BF2*0201‐VL8 binding groove is superimposed onto that of BF2*0401‐IE8. BF2*0201‐VL8 is shown in blue, while BF2*0401‐IE8 is purple. (D, E) Structure‐based amino acid sequence alignment of the α1‐α2 domains of BF2*0201 and BF2*0401, with the secondary structure elements indicated above. Conserved residues are highlighted in red

The 4d0d was solved by 4e0r as a reference model (Figure 1A, ^12^, ^13^). Superposition demonstrates that the structure of the 4d0d molecule^13^ is remarkably similar to that of the 4e0r molecule (Figure 1B), with RMSD between positions of the carbon backbone of A chain of 0.674 Å (Figure 1C), and between positions of α1/α2 domains of 0.550 Å (Figure 1D), and between positions of carbon backbone of the B chain of 0.339 Å (Figure 1E), and an identical orientation of the β2m domains. Although the amino acid sequences of 4e0r and 4d0d have significant differences (Figure 2D, E), the secondary structure of some amino acids in the DSSP of the two is different accordingly. Not only that, but even big differences were apparent in the ratio of the carbon backbone within the two molecules (Figure 1C).

### 3.2 Compared with 4e0r, 4d0d has a relatively broad binding groove

The structures of 4d0d and 4e0r are presented in the form of molecular surfaces. Most of the 4d0d' binding grooves have been covered by the 4e0r', that is to say, pockets A, D, E, and F. Similar to most MHC I molecules but unlike 4d0d, 4e0r has unique pockets A−F (Figure 2A−C).

Pocket A of 4d0d/4e0r is exactly the same. Although the number of residues in pocket B is the same, the types are different. The properties of pocket C and pocket B are the same, and pockets D, E, and F vary both in number and type, especially 4d0d'F pocket has more than one hole to 4e0r', and residues of Tyr110, Met113, Ala114, Tyr115, and Leu157 were found in the peptide‐binding groove of the 4d0d while residues of Ile121 and Phe130 in the 4e0r.

It is the change of spatial configuration caused by these different aa residues that made the binding groove size of 4e0r (1130 Å³)/4d0d (1380 Å³) difference by 250 Å³ in volume (Figure 2A−C). This indicates a significant difference of 4d0d/4e0r, and 4d0d has a relatively broad binding groove, as described by Chappell et al.^12^

### 3.3 Comparison of the properties of the pockets of 4d0d/4e0r peptide‐binding grooves

By analyzing the pockets of 4d0d and 4e0r peptide grooves, we found that the hydrophobic character of pockets A, B, D, E, and F of both 4d0d and 4e0r is dominant, but the pockets C of 4e0r were mainly hydrophilic, while that of 4d0d equally hydrophilic and hydrophobic. About chargeability, pocket A of both is the same without charge, Asp24 of 4d0d in pocket B produces negative charge, while there has no charge in that of4e0r, and Asp73 of 4d0d in pocket C produces negative charge, and all they have in common is a positive charge of Arg9, and Arg152 of 4e0r in pocket D has a positive charge, whereas there is this something that only the public positive charge of Arg in that of 4d0d, the 4e0r in the pocket as well as the public positive charge of Arg9 has both positive and negative charges from Arg111 and Glu149, while is this something that only the public positive charge from Arg in that of 4d0d, and the 4d0d' F pocket owns a positive charge that comes from Arg83 and Lys143, while Arg80, Arg83, and Lys143 that produce positive charges in that of 4e0r.

### 3.4 Specific amino acids in the peptide‐binding groove make the central part of the binding groove of 4d0d extremely broad

**Figure 3:**
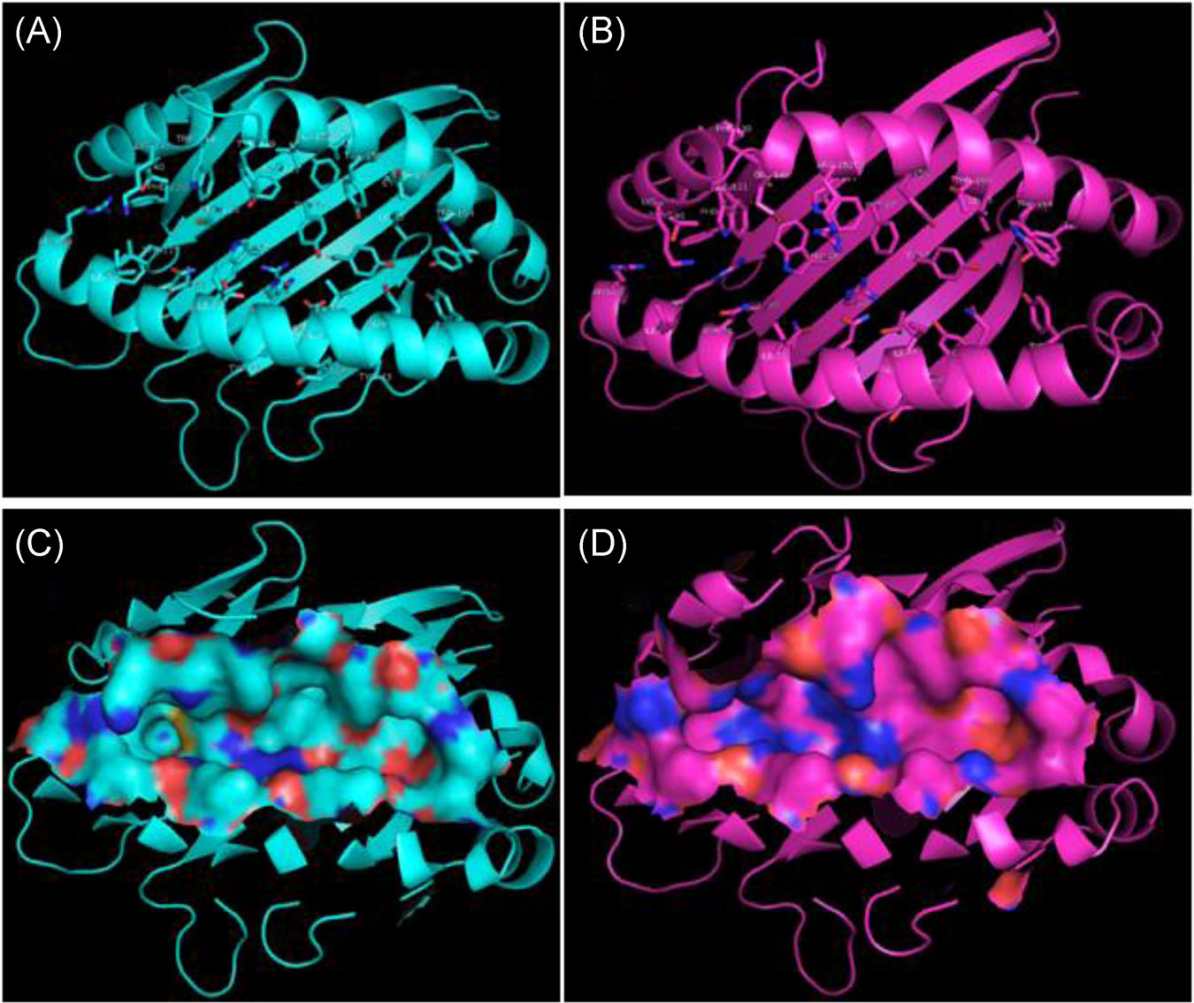
Peptide binding groove amino acid and molecular surface of BF2*0201and BF2*0401. (A) Peptide binding groove amino acid of BF2*0201. (B) Peptide binding groove amino acid of BF2*0401. (C) Peptide binding groove molecular surface of BF2*0201. (D) Peptide binding groove molecular surface of BF2*0401

The 4d0d has Arg9 (positively charged) at the bottom of the groove, with long flexible side chains, as well as the negative Asp24 and Asp73 (Figure 3A), while the 4e0r has charged Arg9 and Arg111 (positively charged) at the bottom of the groove, with long flexible side chains, and the Arg80 in the pocket F at the bottom of the groove (Figure 3B). It is the effect of smaller side‐chain conformation of Tyr111 in 4d0d (Figure 3A) than that of Arg (especially Arg111,) in 4e0r (Figure 3B) that makes the central part of the binding groove of 4d0d peptide broader than that of 4e0r (Figure 3C, D) which can be compounded by that binding groove contained only 6 tyrosine residues (6/37) in 4e0r while 12 tyrosine residues (12/39) in 4d0d.

### 3.5 Compare the peptide presentation motifs of 4d0d/4e0r

The major function of the BF2 molecule is to present antigenic peptides. The more peptides presented, the stronger the resistance of the BF2 molecule. The peptide presentation motifs of BF2*0401 are as follows: x‐(D or E)‐x‐x‐(D or E)‐x‐x‐E.^4^ The eight‐peptide IE8 of 4e0r was consistent with this rule. But the peptide presenting rule of BF2*0201 has not been studied yet. According to the BF2*0201 may be bound to peptide VL8 (VIFPAKSL) or YL9 (YPYLGPNTL),^4^ it is speculated that BF2*0201 can bind an exceedingly large variety of peptides, with relaxed peptide motifs and wide peptide repertoires.

### 3.6 Compare the corresponding relationship between peptide residues and 4d0d/4e0r'pockets

**Figure 4:**
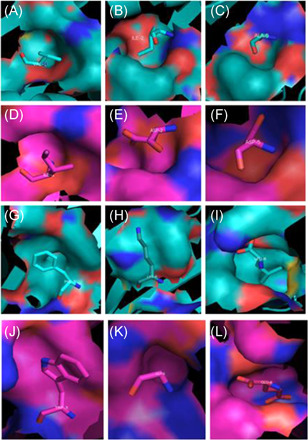
Corresponding relationships of pockets and amino acids in BF2*0201 and BF2*0401, respectively. (A) Pocket A and corresponding amino acids of BF2*0201. (B) Pocket B and corresponding amino acids of BF2*0201. (C) Pocket C and corresponding amino acids of BF2*0201. (D) Pocket A and corresponding amino acids of BF2*0401. (E) Pocket B and corresponding amino acids of BF2*0401. (F) Pocket C and corresponding amino acids of BF2*0401. (G) Pocket D and corresponding amino acids of BF2*0201. (H) Pocket E and corresponding amino acids of BF2*0201. (I) Pocket F and corresponding amino acids of BF2*0201. (J) Pocket D and corresponding amino acids of BF2*0401. (K) Pocket E and corresponding amino acids of BF2*0401. (L) Pocket F and corresponding amino acids of BF2*0401.

The bound antigenic peptides of 4d0d and 4e0r are both 8mer peptides, and the corresponding amino acids of the pockets are different: for 4d0d (Figure 4A−C, G−I), they are P1Val‐pocket A, P2Ile‐pocket B, P5Ala‐pocket C, P3Phe‐pocket D, P6Lys‐pocket E, and P8Leu‐pocket F; while for 4e0r (Figure 4D−F, J−L), they are P1Ile‐pocket A, P2Asp‐pocket B, P5Asp‐pocket C, P3Trp‐pocket D, P6Gly‐pocket E, and P8 Glu‐pocket F, respectively. Due to the difference in the number of peptides, the binding of peptides and pockets does not conform to the rule of human HLA. However, we found that the corresponding amino acid positions of the pockets of 4d0d and 4e0r are the same.

### 3.7 Compare the MHC−peptide interaction of 4d0d and 4e0r

**Figure 5:**
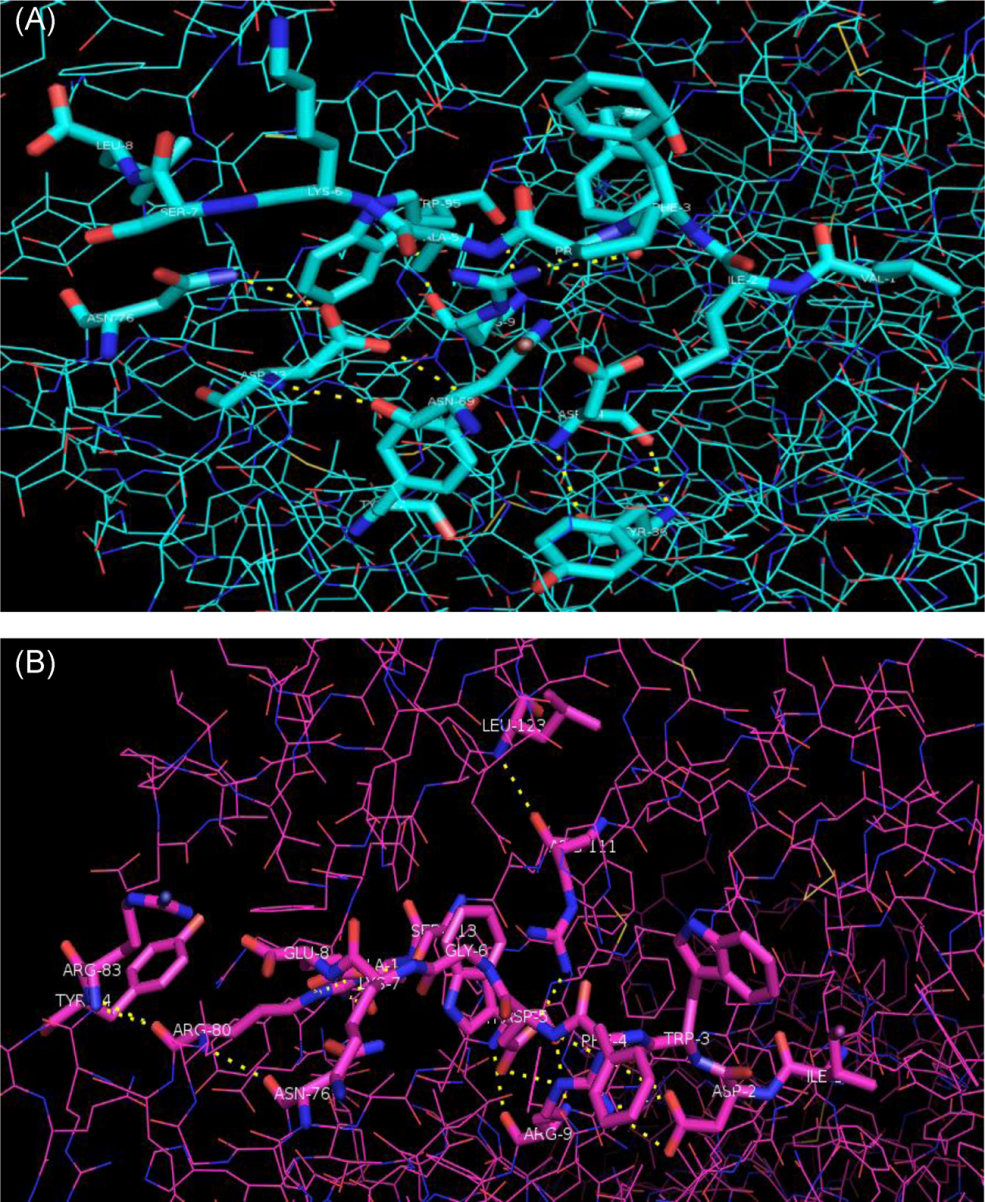
Hydrogen bonds of three important specific amino acids of BF2*0201 and BF2*0401, respectively. (A) The Arg9, Asp24, and Asp73 of BF2*0201 make the groove weak negative charge and, to some extent, tend to combine with the positively charged peptide. (B) The Arg9, Arg80, and Arg111 of BF2*0401 make the groove strong positive charge and extremely tend to combine with the negatively charged peptide.

One salt bond and 14 hydrogen bonds were formed between peptide and binding groove in 4d0d, while in 4e0r 23 hydrogen bonds and 4 salt bonds. The latter had more hydrogen bonds with binding grooves, which may make the binding more firm. The grooves are weakly negatively charged and tend to bind to positively charged peptides to some extent by the Arg9 (positively charged residues), Asp24, and Asp73 (both negatively charged residues) of 4d0d, its Arg9 produced one hydrogen bond with peptide P3Phe, P4Pro peptide bonds, and Tyr97, and itsAsp24 produced two hydrogen bonds with Tyr36, and its Asp73 produced respectively a hydrogen bond with Asn69, Tyr22, and Asn76 (Figure 5A). However, the Arg9, Arg80, and Arg111 (all positively charged residues) of 4e0r make the grooves have a strong positive charge and are extremely easy to bind with negatively charged peptides, its Arg9 produced respectively one hydrogen bond with peptide P3Trp and P4Phe peptide bonds, and produced respectively two hydrogen bonds and two salt bonds with P2Asp, P5Asp peptide bonds, and Trp95, and its Arg80 produced respectively a hydrogen bond with Asn76, Arg83, Tyr84, and Ala114, and produced two hydrogen bonds with Ser113, and produced three hydrogen bonds and salt bonds with P8Glu, and its Arg111 produced one hydrogen bonds and salt bonds with P5 Asp peptide bond and Leu123 (Figure 5B). Thus, although the binding force of the 4e0r molecule is stronger than that of the 4d0d molecule, the selectivity of the peptide is weaker than that of the 4d0d molecule.

## 4 DISCUSSION

The single, dominantly expressed BF2 gene in the chicken is postulated as responsible for the MHC‐related RS or MD resistance in chickens.^4^, ^5^, ^6^ In this study, two points illuminate principles of MHC−peptide interaction, each of which may help in understanding the problems above.

First, the crystal structure 4d0d of the BF2*0201 from the B2 haplotype has two negatively charged residues (Asp24 and Asp73) and one positively charged residue Arg9, pointing upward from the beta chain which forms the base of the binding groove. The resulting groove tends to be both negative charge and positive charge, which should explain both positive charge and negative charge peptide motif that can be determined by B2 haplotype, while the 4e0r of B4 haplotype exists as a highly positively charged surface that has not so far been observed in other MHC molecules,^3^, ^13^ which has unique positive charge groove to bind some negative charge anchor residues. Due to the amino acid difference between the two binding grooves, the size and chargeability of the binding groove of them are different, leading to different selections of binding peptides.

Second, there are the shorter side chain Arg111 in the 4d0d peptide‐binding groove of BF2*0201 than side chain Tyr111 in the 4e0r peptide‐binding groove of BF2*0401,^13^ and there are some differences of specific amino acids between Arg9, Arg80, and Arg111 in the 4e0r peptide‐binding groove of BF2*0401^13^ and Arg9, Asp24, and Asp73 in the 4d0d peptide‐binding groove of BF2*0201. Therefore, the middle of the binding groove of BF2*0201 becomes obviously wider and the bound restriction of amino acid becomes less.

In this study, the configuration of BF2*0201 that leads to a relatively promiscuous motif was focused on,^5^, ^12^ presenting peptides to T cells on the cell surface, compared with that of BF2*0401.^13^ Thus, the BF2 of the B2 usually presents more peptides than that of the B4, leading to resistance to pathogens, for example, Rous Sarcoma virus and Marek's Disease Virus. In previous literature,^11^ there are generally more peptides presented by the BF2 of B21 than by that of B2 haplotypes, so B21 has stronger host antiviral ability than B2 haplotype. This is basically consistent with results—B21 haplotypes have the strongest resistance to MD, B2 medium resistance, and B4 weak resistance,^7^, ^10^, ^11^ and B21, and B2 haplotypes confer stronger resistance to RS than B4.^5^, ^9^, ^10^

## CONFLICT OF INTERESTS

The authors declare that there are no conflict of interests.

## AUTHOR CONTRIBUTIONS

*Conceptualization*: Yuan‐chang Jin, Yu‐feng Li, and Wei Wang. *Methodology*: Yu‐feng Li and Wei Wang. *Software*: Yuan‐chang Jin. *Validation*: Li‐xia Jiang, Yu‐jie Wu, and Juan Dai. *Formal analysis*: Mei‐lin Hao and Jing‐fen Chen. *Data curation*: Gang Zeng. *Writing—original draft preparation*: Chuan‐dan Zheng and Min‐min Yu. *Writing—review and editing*: Ming‐li Chen and Bo‐ping Zeng. *Supervision*: Yuan‐chang Jin.

## Data Availability

The data that support the findings of this study are available on request from the corresponding author. The data are not publicly available due to privacy or ethical restrictions.

## References

[1] Kelly A , Trowsdale J . Genetics of antigen processing and presentation. Immunogenetics. 2019;71(3):161‐170. 30215098 10.1007/s00251-018-1082-2 PMC6394470

[2] Kim T , Hunt HD , Parcells MS , van Santen V , Ewald SJ . Two class I genes of the chicken MHC have different functions: BF1 is recognized by NK cells while BF2 is recognized by CTLs. Immunogenetics. 2018;70(9):599‐611. 29947944 10.1007/s00251-018-1066-2

[3] Kim T , Hunt HD , Parcells MS , van Santen V , Ewald SJ . Correction to: Two class I genes of the chicken MHC have different functions: BF1 is recognized by NK cells while BF2 is recognized by CTLs. Immunogenetics. 2018;70(10):693‐694. 29982918 10.1007/s00251-018-1069-z

[4] Koch M , Camp S , Collen T , et al. Structures of an MHC class I molecule from B21 chickens illustrate promiscuous peptide binding. Immunity. 2007;27(6):885‐899. 18083574 10.1016/j.immuni.2007.11.007

[5] Wallny HJ , Avila D , Hunt LG , et al. Peptide motifs of the single dominantly expressed class I molecule explain the striking MHC‐determined response to Rous sarcoma virus in chickens. Proc Natl Acad Sci USA. 2006;103(5):1434‐1439. 16432226 10.1073/pnas.0507386103 PMC1360531

[6] Sherman MA , Goto RM , Moore RE , Hunt HD , Lee TD , Miller MM . Mass spectral data for 64 eluted peptides and structural modeling define peptide binding preferences for class I alleles in two chicken MHC‐B haplotypes associated with opposite responses to Marek's disease. Immunogenetics. 2008;60(9):527‐541. 18612635 10.1007/s00251-008-0302-6 PMC3339847

[7] Parker MA , Schierman LW . Suppression of humoral immunity in chickens prevents transient paralysis caused by a herpesvirus. J Immunol. 1983;130(5):2000‐2001. 6300237

[8] Dalgaard TS , Hojsgaard S , Skjodt K , Juul‐Madsen HR . Differences in chicken major histocompatibility complex (MHC) class Ialpha gene expression between Marek's disease‐resistant and ‐susceptible MHC haplotypes. Scand J Immunol. 2003;57(2):135‐143. 12588659 10.1046/j.1365-3083.2003.01207.x

[9] Bacon LD , Witter RL , Crittenden LB , Fadly A , Motta J . B‐haplotype influence on Marek's disease, Rous sarcoma, and lymphoid leukosis virus‐induced tumors in chickens. Poult Sci. 1981;60(6):1132‐1139. 6267578 10.3382/ps.0601132

[10] Aeed PA , Collins WM , Briles WE , Zsigray RM . Influence of different B‐complex recombinants on the outcome of Rous sarcomas in chickens. Anim Genet. 1993;24(3):177‐181. 8395777 10.1111/j.1365-2052.1993.tb00283.x

[11] Jin Y , Wang W , Yu M , et al. Study on the contrast of the MHC‐peptide interaction of B2/B21 haplotype and MHC‐related virus resistance in chickens. Immun Inflamm Dis. 2021;9(4):1670‐1677. 34473901 10.1002/iid3.520 PMC8589374

[12] Chappell P , Meziane el K , Harrison M , et al. Expression levels of MHC class I molecules are inversely correlated with promiscuity of peptide binding. eLife. 2015;4:e05345. 25860507 10.7554/eLife.05345 PMC4420994

[13] Zhang J , Chen Y , Qi J , et al. Narrow groove and restricted anchors of MHC class I molecule BF2*0401 plus peptide transporter restriction can explain disease susceptibility of B4 chickens. J Immunol. 2012;189(9):4478‐4487. 23041567 10.4049/jimmunol.1200885 PMC5018395

